# The Impact of Nationwide Education Program on Clinical Practice in Sepsis Care and Mortality of Severe Sepsis: A Population-Based Study in Taiwan

**DOI:** 10.1371/journal.pone.0077414

**Published:** 2013-10-04

**Authors:** Yu-Chun Chen, Shih-Chieh Chang, Christy Pu, Gau-Jun Tang

**Affiliations:** 1 Department of Medical Research and Education, National Yang-Ming University Hospital, Yilan, Taiwan; 2 Institute of Hospital and Health Care Administration, School of Medicine, National Yang-Ming University, Taipei, Taiwan; 3 Division of Chest Medicine, Department of Internal Medicine, National Yang-Ming University Hospital, Yilan, Taiwan; 4 Faculty of Medicine, School of Medicine, National Yang-Ming University, Taipei, Taiwan; D'or Institute of Research and Education, Brazil

## Abstract

**Objectives:**

We investigated the effect of a nationwide educational program following surviving sepsis campaign (SSC) guidelines. Physicians’ clinical practice in sepsis care and patient mortality rate for severe sepsis were analyzed using a nationally representative cohort.

**Methods:**

Hospitalizations for severe sepsis with organ failure from 1997 to 2008 were extracted from Taiwan’s National Health Insurance Research Database (NHIRD), and trends in sepsis incidence and mortality rates were analyzed. A before-and-after study design was used to evaluate changes in the utilization rates of SSC items and changes in severe sepsis mortality rates occurred after a national education program conducted by the Joint Taiwan Critical Care Medicine Committee since 2004. A total of 39,706 hospitalizations were analyzed, which consisted of a pre-intervention cohort of 14,848 individuals (2000-2003) and a post-intervention cohort of 24,858 individuals (2005-2008).

**Results:**

The incidence rate of severe sepsis increased from 1.88 per 1,000 individuals in 1997 to 5.07 per 1,000 individuals in 2008. The cumulative mortality rate decreased slightly from 48.2% for the pre-intervention cohort to 45.9% for the post-intervention cohort. The utilization rates of almost all SSC items changed significantly between the pre-intervention and post-intervention cohorts. These changes of utilization rates were found to be associated with mild reduction in mortality rate.

**Conclusion:**

The nationwide education program through a national professional society has a significant impact on physicians’ clinical practice and resulted in a slight but significant reduction of severe sepsis mortality rate.

## Introduction

Sepsis is the eleventh leading cause of death in the Taiwan. The incidence of sepsis has increased over the past decades and its mortality rates have ranged between 30 to 50% globally [1-4]. Over the past decade there have been a host of discoveries about sepsis in the areas of pathogenesis, prevention and therapeutic strategies [5,6]. Numerous clinical trials have demonstrated implementation of multiple evidence-based clinical practice protocols as a bundle based on the Surviving Sepsis Campaign (SSC) guidelines, either through an education program or via strict protocol control, is able to improve patient outcomes [7-11].

Education is able to change the behavior of physicians and has a positive impact on the outcomes of critically ill patients [12-15]. Following the publication of the surviving sepsis campaign in 2004 [16], a nationwide educational program was launched by the joint Taiwan Critical Care Medicine Committee. This committee consists of three medical societies, namely Critical Care Medicine, Emergency and Critical Care Medicine, and Pulmonary and Critical Care Medicine (total board certified specialists = 2102). Briefly, the education program consisted of at least 10-hours of training sessions for each intensivist. A Taiwanese intensivist was required to complete the training program to take critical care license examinations and to extend their licenses. The education program has been held at least 5 times a year since 2004 and almost all physicians who work in an ICU have completed the training course.

The aim of our study was to evaluate the effect of this SSC-based educational program on the outcome of severe sepsis patients. We used Taiwan’s National Health Insurance Research Database (NHIRD), which contains health care data of virtually all residents in Taiwan, to assess the incidence and mortality rate for severe sepsis from January 1, 1997 to December 31, 2008. We identified the change in the utilization rate of items in SSC after the implementation of this nationwide education program and examined whether these changes had any impact on outcomes among sepsis patients.

## Materials and Methods

### Data source

Taiwan’s NHIRD is a publically-released and de-identified research database that virtually covers all the population in Taiwan (coverage rate, 99.6% during 2011). Within Taiwan’s national health insurance (NHI) scheme, medical claims are sent to the Bureau of National Health Insurance (BNHI) of Taiwan for cross-checking and validation with the aim of ensuring the accuracy of diagnosis coding. Hospitals that are involved in fraudulent coding, overcharging or malpractice are subjected to heavy penalties or suspension. A recent validation study supports the reliability of the NHIRD diagnostic codes [17]. In order to help ensure privacy, the National Health Research Institute (NHRI) recompiles claims data and makes the data publicly available for researchers in Taiwan. Individual and hospital identifiers are unique to the research database and cannot be used to trace individual patients or health service providers [18]. The regulations for studies using such anonymized claims databases were just proposed when we conducting the current study. Before the enforcement of the regulations, a claim-based study was not required to submit to a full review by the Institutional Review Board if it was considered as not infringing ethical issues. Our study was approved as (NHIRD #101006). Based on the above, the present study is exempted from full review by the Institutional Review Board.

### Study cohort

To investigate the trend in incidence and mortality rates for severe sepsis, we extracted all hospitalizations for severe sepsis with organ failure during the period 1997 to 2008 using the nationwide inpatient expenditure files (DD) of the NHIRD. The database includes all hospitalizations occurred in Taiwan. Each record of hospitalization is comprehensive and includes up to five discharge diagnosis codes and five main procedure codes as well as discharge status; because of this pre-hospitalization comorbidities and hospitalization severity are able to be accurately measured. However, the details of each admission, such as the use of different medications and procedures are not included in this data file and thus other NHIRD datasets were used for the remaining parts of the analysis.

To compare changes in utilization rates of relevant intervention in sepsis care and any associated change in mortality rates before and after the implementation of the SSC program in 2004, a before-after study design was used. To do so, two randomly sampled study cohorts were extracted from the NHIRD databases. The first cohort consisted of one million randomly sampled individuals, who are representative of the Taiwan population in 2000; hereafter this is called the LHID2000 cohort. A second cohort, using a similar procedure, was used to select another one million subjects who were representative of the Taiwan population in 2005 (thereafter, the LHID2005 cohort). Both cohorts were followed for four years (2000~2003 for LHID2000 and 2005~2008 for LHID2005) (Figure S1). The NHRI has reported no significant differences in age, sex, or healthcare costs between these cohorts and the population as a whole under the NHI program. LHID2000 and LHID2005 contain all of the detailed medical claims under Taiwan’s NHI, and these were then used to compare the incidence and mortality rate for severe sepsis before and after the implementation of the SSC program in 2004. Patients who were hospitalized for severe sepsis were then extracted from both cohorts. The pre-intervention cohort (LHID2000 cohort) included 14,848 hospitalizations for severe sepsis with organ failure during the period 2000~2003, which is the four years before the implementation of the SSC program. Similarly a post-intervention cohort (LHID2005 cohort) was created with 24,858 sepsis hospitalizations during 2005-2008, which is the four years after the implementation.

### Identification of hospitalizations with severe sepsis

Hospitalizations with severe sepsis were identified using the ICD-9-CM codes for both bacterial or fungal infectious processes (Table S1) and a diagnosis that is indicative of acute organ dysfunction (Table S2). This approach was proposed by Angus et al [19] and modified by Shen et al [20] in order to better fit with Taiwan’s NHIRD system. A validation test has shown a high agreement between expert medical chart review and this approach [20].

### Ascertainment of covariates and hospital mortality

The date of incidence of severe sepsis was designated as the first date of each patient’s specific hospitalization. If there was any subsequent re-hospitalization at the same hospital within 7 days after discharge, this was regarded as a continuation of the previous admission. Death within 30 days after discharge was regarded as hospital morality. The Charlson comorbidity index was calculated using the diagnostic codes from the outpatient records and discharge codes from the hospitalization records 1 year before each admission.

### Determination of the utilization rates for SSC items

Both medical procedures and medications during hospitalization can be completely identified from the database. The use of the eighteen SSC items as recommended by the program was determined using the claims records for procedures and medication used during the hospitalization. The procedures included use of central venous catheterization (CVP), blood transfusion, measurement of blood lactate level, diagnostic culture, use of a ventilator and use of hemodialysis. Measurement of weaning parameters was deemed as weaning attempts. A procedure was coded 1 if used, and 0 otherwise.

Any exposure to medications, such as the use of broad spectrum antibiotics, systemic steroids (ATC code: H02AB), vasopressor agents (dopamine and norepinephrine), rhAPC, muscle relaxants (M03), sodium bicarbonate (B05XA02), H2-receptor antagonists (A02BA) and proton pump inhibitors (PPI, A02BC) during the hospitalization were also identified.

### Estimation of the reduction in hospital mortality after implementation of the SSC program

To estimate the impact on hospital mortality for utilizations of the SSC items, hospital mortality changes associated with changes in utilization rate for each SSC item between the pre-intervention cohort and post-intervention cohort were calculated. The utilization rates of the SSC items as well as the mortality rates for severe sepsis were summarized at the hospital level. These results were weighted by the frequency of admission, the behavior change and hospital mortality rate then adjusted for age, sex, co-morbidities and numbers of organ failure. The results were then compared across hospitals.

### Statistical analysis

All of the data were linked using a SQL server 2008 (Microsoft Corp.) and analyzed using STATA 12 software (StataCorp LP, College Station, Texas). The care of severe sepsis with organ failure that was affected by the SSC program was assumed to be a residual of a Poisson regression model. The annual risk-adjusted expected mortality was then estimated using the fitted Poisson regression model, holding the residual constant. Differences between the pre-intervention cohort and post-intervention cohort were then compared using t-tests and chi-square tests.

Poisson regression models were used to estimate the difference in utilization rate of each item as recommended by the SSC guidelines (SSC items) between the pre-intervention cohort and post-intervention cohort. A specific poisson regression was fitted for each SSC item, and for each regression a dummy variable was used to indicate the different cohorts (the dummy variable was coded 0 for the pre-intervention cohort and 1 for the post-intervention cohort). The difference in utilization rate of each SSC item was determined using the coefficient estimated for this dummy variable.

We then estimate the reduction in mortality associated with the difference in utilization rate of SSC items. Since a hospital may be present in both cohorts, generalized estimating equation (GEE) models were used to account for repeated measurement. Unstructured correlation and an identity link function were assumed. All models were controlled for patient age, sex, co-morbidities and the number of organ failures. A two-tailed *p*=0.05 was considered statistically significant.

## Results

### Annual incidence rates and mortality rates for severe sepsis in Taiwan

**Figure 1:**
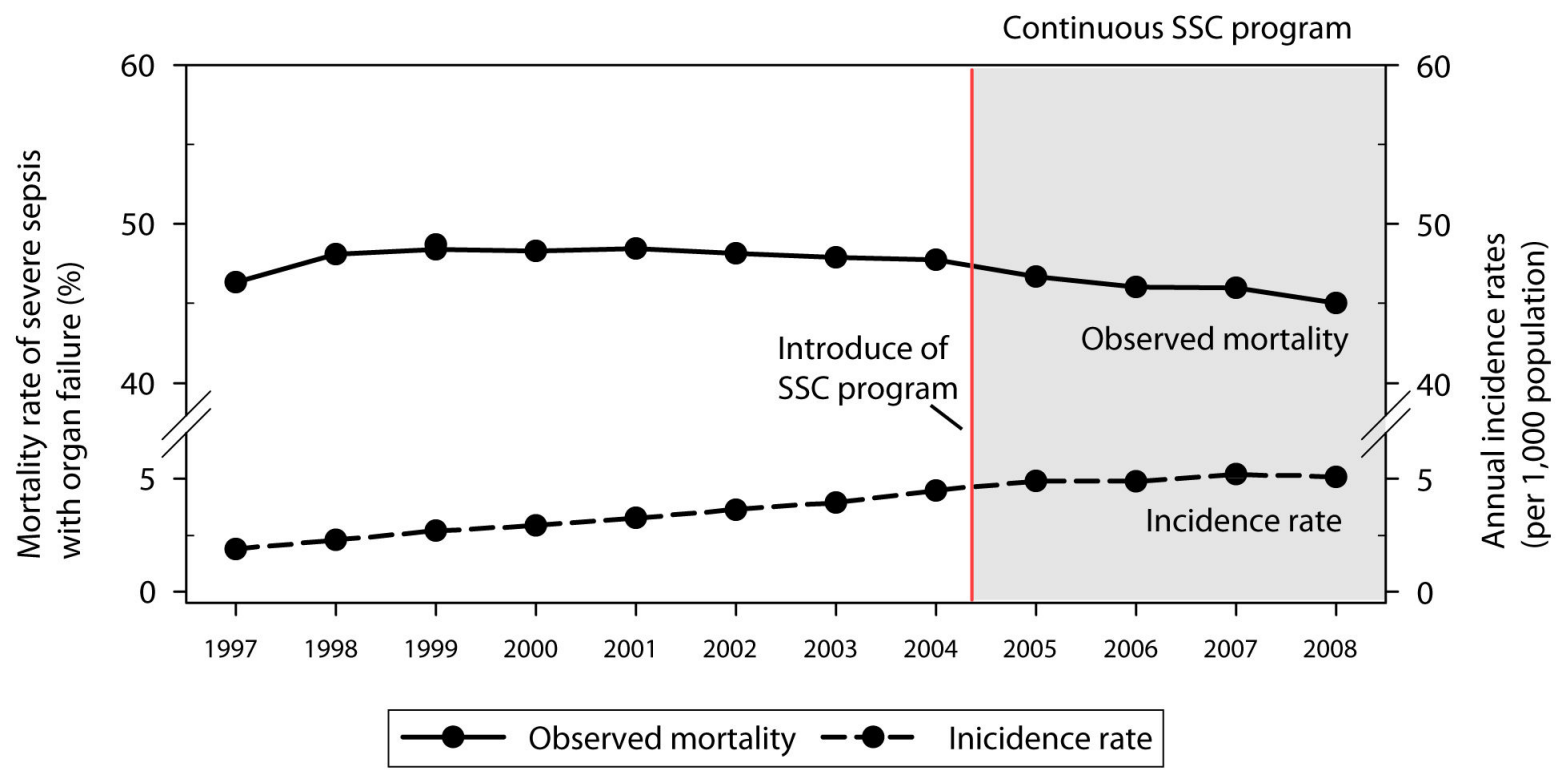
Annual incidence rates and observed mortality rates for hospitalizations with severe sepsis with organ failure between 1997 and 2008 in Taiwan. (The nationwide SSC program was launched in 2004.)

The incidence rates for hospitalization with severe sepsis with organ failure have increased gradually with time (Figure 1). There has been a more than two fold increase (incidence rate ratio, 2.67; 95% confidence interval, 95% C.I., 2.67-2.73) in the crude incidence rate over 13 years (1.88/1,000 individuals in 1997 vs. 5.07/1,000 individuals in 2008, *p*<0.001). However, the observed mortality rates for severe sepsis have decreased steadily from 46.3% in 1997 and 48.2% in 1998 to 45.0% in 2008 (*p*<0.001). A mild difference in the cumulative mortality rates before and after the year 2004 (cumulative mortality rate during 2000-2003 vs. 2005-2008, 48.2% vs. 45.9%, *p*<0.001) can be observed. Moreover, the average annual percentage change (AAPC) in mortality rate was increased by -1.2% after the launch of the nationwide SSC program in 2004 (AAPC in 2000-2003 vs. 2005-2008, -0.3% vs. -1.5%, *p*<0.001) (Table 1).

**Table 1:** Annual incidence rates, observed mortality rates and annual percentage change of mortality rates for hospitalizations with severe sepsis with organ failure between 1997 and 2008 in Taiwan.

Year	No. of severe sepsis with organ failure	Annual incidence rate (per 1,000)	Observed mortality rates (%)	Annual percentage change of mortality rates (%)
1997	40,856	1.88	46.3	−
1998	50,094	2.28	48.1	3.8
1999	59,181	2.68	48.4	0.7
2000	64,999	2.92	48.3	-0.2
2001	72,762	3.25	48.5	0.3
2002	81,530	3.62	48.2	-0.6
2003	88,712	3.92	47.9	-0.6
2004	101,511	4.47	47.7	-0.3
2005	110,927	4.87	46.7	-2.2
2006	111,139	4.86	46.0	-1.4
2007	119,006	5.18	46.0	-0.2
2008	116,749	5.07	45.0	-2.1

### Comparison of the demographics and utilization rates in SSC items between the pre-intervention and post-intervention cohorts

The post-intervention cohort had a more advanced age (mean age, 63.8 vs. 65.8, *p*<0.001), more comorbidities (mean Charlson index score, 3.92 vs. 3.46, *p*<0.001) and higher severity of sepsis (mean number of organ failures, 1.31 vs. 1.26, *p*<0.001) than the pre-intervention cohort. To make this study comparable to other studies, we used the list of diagnosis to identify sepsis related hospitalization as Angus et al and Shen et al [19,20]. A minority (less than 3%) of diagnosis codes were not necessarily related to sepsis. However, there was no significant difference between the groups. Moreover, a Kolmogorov-Smirnov test suggested the equality of distribution of diagnosis between pre-intervention and post-intervention cohort (P=0.767) (Table S3).

**Figure 2:**
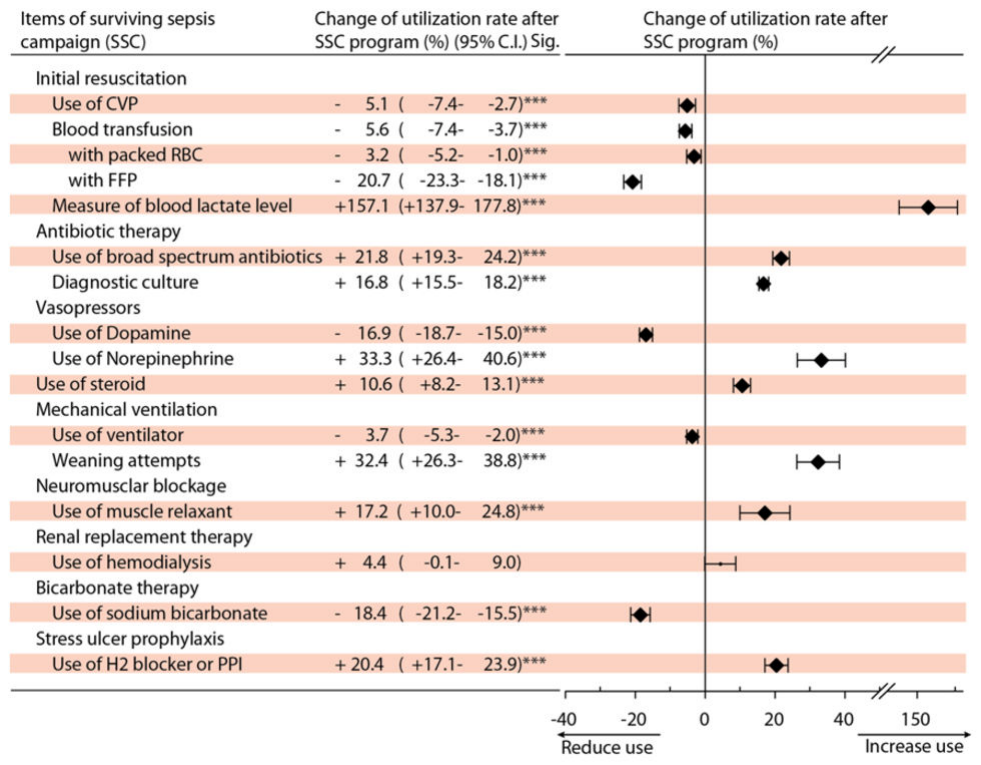
Utilization rate changes for items in the SSC guidelines before and after the SSC program was implemented in Taiwan. (pre-intervention cohort (2000-2003, before SSC program) vs. post-intervention cohort (2005-2008, after SSC program), n=39,706).

Most SSC items showed a significant change between the two cohorts and these were mostly an increased in the utilization rate between the two cohorts (Table 2). Figure 2 shows the percentage change for the differences in the utilization rates of SSC items between the two cohorts after controlling for the age, sex, severity and comorbidities of the patients.

**Table 2:** Characteristics, utilization rates of items of the surviving sepsis campaign (SSC) and outcomes are compared between before implementation of the SSC program (pre-intervention cohort, 2000-2003) and after implementation of the SSC program (post-intervention cohort, 2005-2008).

					Pre-intervention cohort (2000-2003)		Post-intervention cohort (2005-2008)			
					(n=14,848)		(n=24,858)			
	Characteristics				n	(%)	n	(%)	*p* value	Sig.
	Gender								0.547	
		Male			8810	(59.3)	14673	(59.0)		
		Female			6038	(40.7)	10185	(41.0)		
	Age								<0.001	***
		80-			3707	(25.0)	7985	(32.1)		
		60-79			7066	(47.6)	10616	(42.7)		
		40-59			2514	(16.9)	4174	(16.8)		
		20-39			877	(5.9)	1173	(4.7)		
		0-19			684	(4.6)	910	(3.7)		
	Charlson Index								<0.001	***
		5-			4596	(31.0)	9140	(36.8)		
		3-4			3417	(23.0)	5977	(24.0)		
		1-2			4787	(32.2)	6943	(27.9)		
		0			2048	(13.8)	2798	(11.3)		
	No. of organ dysfunction								<0.001	***
		4+			40	(0.3)	72	(0.3)		
		3			520	(3.5)	1093	(4.4)		
		2			2715	(18.3)	5329	(21.4)		
		1			11573	(77.9)	18364	(73.9)		
	Organ dysfunction									
		Cardiovascular			3163	(21.3)	5485	(22.1)	0.075	
		Hematologic			647	(4.4)	965	(3.9)	0.020	*
		Hepatic			1218	(8.2)	1712	(6.9)	<0.001	***
		Metabolic			156	(1.1)	286	(1.2)	0.359	
		Neurologic			913	(6.1)	1614	(6.5)	0.174	
		Renal			3528	(23.8)	6647	(26.7)	<0.001	***
		Respiratory			9098	(61.3)	15886	(63.9)	<0.001	***
Utilization rates of items of surviving sepsis campaign										
	Initial resuscitation									
		Use of CVP			5733	(38.6)	9482	(38.1)	0.355	
		Blood transfusion			7610	(51.3)	12583	(50.6)	0.222	
			with packed RBC		6758	(45.5)	11500	(46.3)	0.148	
			with FFP		4331	(29.2)	6022	(24.2)	<0.001	***
		Measure of blood lactate level			720	(4.8)	3155	(12.7)	<0.001	***
	Antibiotic therapy									
		Use of broad spectrum antibiotics			6812	(45.9)	14251	(57.3)	<0.001	***
		Use of diagnostic culture			10460	(70.4)	20723	(83.4)	<0.001	***
	Vasopressors									
		Use of Dopamine			6417	(43.2)	9513	(38.3)	<0.001	***
		Use of Norepinephrine			1508	(10.2)	3617	(14.6)	<0.001	***
	Use of steroid				6404	(43.1)	12070	(48.6)	<0.001	***
	Recombinant human activated protein C									
		Use of activated protein C			0	(0.0)	16	(0.1)	<0.001	***
	Mechanical ventilation									
		Use of ventilator			8262	(55.6)	13848	(55.7)	0.900	
		Weaning attempts			2063	(13.9)	4644	(18.7)	<0.001	***
		Mean duration of ventilation ± SE			15.1	± 0.4	14.8	± 0.3	0.122	
	Neuromuscular blockage									
		Use of muscle relaxant			1340	(9.0)	2553	(10.3)	<0.001	***
	Renal replacement therapy									
		Use of hemodialysis			2329	(15.7)	4329	(17.4)	<0.001	***
	Bicarbonate therapy									
		Use of sodium bicarbonate			3711	(25.0)	5390	(21.7)	<0.001	***
	Stress ulcer prophylaxis									
		Use of H2 blocker or PPI			4634	(31.2)	9651	(38.8)	<0.001	***

(n=39,706).

SE: standard error. NTD: New Taiwan Dollar. CVP: central venous catheterization. FFP: frozen fresh plasma. PPI: proton pump inhibitor

Sig.: *p* < 0.05: * *p*<0.01: ** *p*<0.001: ***

The SSC item with the greatest significant increase in utilization rate by the post-intervention cohort was measurement of the patient’s lactate level (+157.1%), followed by the use of norepinephrine (+33.3%), the number of weaning attempts (+32.4), the use of broad spectrum antibiotics (+21.8%), the use of H2 blockers or PPI (+20.4%), the use of a muscle relaxant (+17.2%) and the use of a diagnostic culture (+16.8%). In contrast, blood transfusion with fresh frozen plasma (FFP) showed the greatest reduction (-20.7%), followed by use of sodium bicarbonate (-18.4%) and use of dopamine (-16.9%).

### Reduction in hospital mortality rate after SSC program

**Figure 3:**
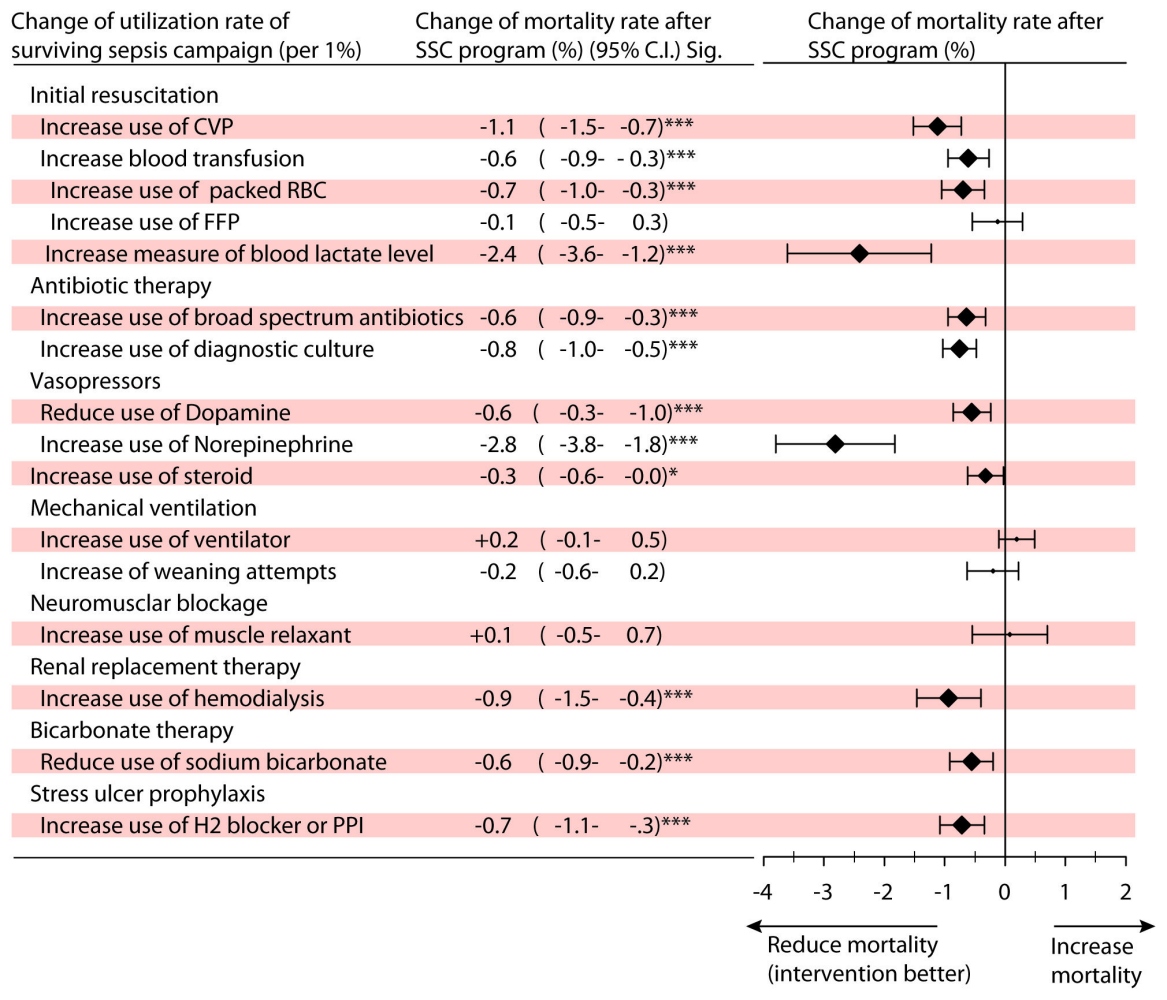
Effects of utilization rate changes for items in the SSC guidelines on changes in mortality rates in Taiwan (pre-intervention cohort (2000-2003, before SSC program) vs. post-intervention cohort (2005-2008, after SSC program), n=39,706).

The change in utilization rates of SCC items may be associated with a mild change in mortality rates. After fitting a multivariate regression model that controlled for age, sex, comorbidities and severity of the patients, the change in mortality rate at a specific hospital corresponding to the per unit change in utilization rate of each SSC items were explored and are shown in Figure 3.

For the majority of the SSC items, the change in utilization rate was significantly associated with a small reduction in mortality rate. This suggests that the change in compliance with SSC guidelines may have a positive effect on the hospital mortality rate (Figure 3). Two items including “increase use of norepinephrine” and “increase measure of blood lactate level” had the largest effects on mortality rates. In Figure 3, one unit increase measure of blood lactate level would lead to a reduction of mortality rate at 2.4%. The finding suggested that if a hospital increased the use of measure of blood lactate level at 1%, the severe sepsis related mortality rate would reduce for 2.4%.

For the use of vasopressors, “increase use of norepinephrine” leads to a mortality rate reduction at 2.8% while “reduce use of dopamine” leads to a mortality rate reduction at 0.6%. The finding implied if a hospital reduces use of dopamine in addition to increase use of norepinephrine, the mortality rate reduction would reduce for more 0.6% from 2.8%.

However, four SSC items, namely an increase of FFP, an increased use of ventilators, an increase in weaning attempts and an increase use of muscle relaxant, showed no significant association with change in mortality rate.

## Discussion

A national educational program run by the national professional societies changed physicians’ clinical behavior in septic patient care and may be associated with a slight but statistically significant reduction in the mortality rate of severe sepsis. The incidence and mortality rate for severe sepsis differ across different areas and also vary with time. Our study found that the incidence rate for sepsis hospitalization increased by 2.7 fold from 1997 to 2008. Nevertheless, hospital mortality rate declined gradually with time. The utilization rates of almost all (17 out of 18) SSC items changed significantly after 2004. Moreover, the change in utilization rates was found to be significantly associated with a mild reduction in mortality rates. Our findings suggest that a nationwide education program like the one instituted in Taiwan is able to positively change physician behavior in sepsis care following the SSC guidelines and may have some benefits in patient outcome for severe sepsis.

Our findings are compatible with worldwide trends. In the United States, the National Hospital Discharge Survey found that while there was an increased incidence of sepsis (defined by the code for septicemia), there was also a reduction in the disease-related in-hospital mortality rate from 27.8% (1979 to 1984) to 17.9% (1995 to 2000) as the incidence rate increased from 0.83 (1997) to 2.40 (2000) per 1,000 population [3]. When severe sepsis is examined, US state hospital discharge records (n= 5 6,621,559) showed a national incidence of 3.0 cases per 1,000 population with a mortality rate of 28.6% in 1995 [19]. Discharge data from the Nationwide Inpatients Sample showed that the hospital rate had doubled from 1993 to 2003 (0.65 to 1.35 per 1,000 population), while the hospital mortality rate fell from 45.8% to 37.8% [2]. In Taiwan, the cumulative mortality for severe sepsis was 48.2% from 2000 to 2003 and this had decreased to 45.9% from 2005 to 2008 (Table 1). It might be argued that observed mortality rate for severe sepsis in Taiwan had started to decline from 2002 onwards (Figure 1). From 2000 to 2002, several major advances, such as early goal-directed therapy (EGDT) [21], recombinant human activated protein C [6] and corticosteroid therapy [22], were introduced as part of the treatments used for severe sepsis and these may have produced some improvement in survival. The average annual percentage changes in mortality rates were -1.5% for the post-SSC cohort and -0.3 for the pre-SSC cohort, there was a slight but statistically significant difference in annual mortality rate for severe sepsis after the SSC implementation.

The nation wide surviving sepsis campaign was launched since 2004 and covered almost all physicians who care for ICU patients. Our data support the idea that the implementation of the SCC program changed the physician behavior in septic care and may be associated with declining severe sepsis mortality. A multi-national study that included 15022 patients evaluated the effects of this international guideline-based performance improvement program and found that the unadjusted hospital mortality rate had decreased from 37.0% in 2005 to 30.8% in 2008 [23]. Studies done in Spain found that installation of a hospital-wide quality improvement program reduced the in-hospital mortality rate from 57.3% (2004-5) to 37.5% (2005-8), while the introduction of a national educational program changed hospital mortality from 44.0% (2005) to 39.7% (2006) [12,23]. In contrast to the Spanish study [12], which prospectively designed the study and systemically organized the education program for physicians and nurses, and the study in the US that strictly implemented the protocol [11] or standardized the order set [10], the intervention in Taiwan is an educational program run by a national society. The implementation of the guidelines is not related to either a positive financial incentive or to punishment for not following the guideline. Even with the limitations associated with an education program, we were still able to demonstrate significant behavior changes in process-of-care and a small overall reduction in mortality.

The uniqueness of our findings lie in the fact that we used a sufficiently long observation period of up to four years and this allowed the sustained effect on mortality reduction to be demonstrated.

Similar to the previous Spanish studies [12], the major changes in item utilization that occurred after introduction of the educational program included increased utilization in lactate measurement, more diagnostic cultures, greater used of broad-spectrum antibiotics and greater use of low-dose steroids.

We found that measurement of lactate and use of norepinephrine had most significant positive effects on survival. The use of serial measurements of serum lactate as part of the sepsis resuscitation bundle is strongly associated with an improved outcome [24]. Although serum lactate measurements across our patients are relatively infrequent, utilization increased significantly after our national education program (4.8% vs. 12.7%), and this increase in the measurement of lactate levels across the post-intervention cohort improved survival. Indeed, we found that there is still much room for improvement in sepsis care in Taiwan.

Dopamine or norepinephrine is recommended in SSC guidelines as the first-line vasopressor when treating septic shock. A systematic review of six randomized clinical trials has shown that norepinephrine is a better vasopressor choice than dopamine when treating septic shock [25]. In addition, it has been found that dopamine increases the risk of arrhythmia when compared with norepinephrine [25-27]. Our analytical results agree with this earlier study and show that replacing dopamine with norepinephrine improves survival [25].

Increased use of central venous catheterization, increased use of diagnostic cultures, greater use of broad-spectrum antibiotics, greater use of corticosteroids, higher levels of renal replacement therapy, more stress ulcer prophylaxis and reduced use of sodium bicarbonate also resulted in significant though small decreases in mortality. Although SSC guidelines suggest avoiding neuromuscular blockers if possible, small increased muscle relaxant use in post-education cohort did not affect the final outcome in our study. For patients with acute respiratory distress syndrome, neuromuscular blockers may improve gas exchange and ventilator-induced lung injury, even final outcome[28].

Several limitations that affect our study should be noted. Firstly, APACHE II, which is routinely used to evaluate the severity of critically ill patients, was unavailable. Moreover, either systemic bias or selection may exist because of the quality of diagnostic codes. As the chart level data were not available in the NHIRD, we used both diagnostic codes (for infectious process) and procedure codes (for organ failure) to ascertain the diagnosis of severe sepsis. Although such issues should be minimal using a sophisticate, validated algorithm for NHIRD [20], the increased incidence of severe sepsis through the years is likely contributed to changes in documentation and coding of sepsis and organ dysfunction due to increased awareness.

Secondly, no control group is available to validate the effectiveness of SSC program due to the nature of the training. Indeed, the improvement of health care is a multi-factorial process that requires quite amount of effort from various input. In our study, we can’t demonstrate differences between the groups regarding several important issues in general critical care such as nutritional support and prevention of nosocomial infection. We thus used a before and after study design that compared the utilization rates of severe sepsis items after the intervention and, moreover, we correlated the change of utilizations rate reduction in mortality rates. These findings suggested that the most likely explanation for the change is the education program itself. However, we cannot eliminate temporal effect given the reduction in sepsis related crude mortality is small. The impact of the program might be overemphasized and required further study.

Thirdly, information on the exact timing of each procedure is not available from the NHIRD and this prevents further analysis of various time-relevant SSC items including EGDT and the optimal time to administer broad-spectrum antibiotics. The absence of this information will allow us to only investigate the utilization rate but not the compliance with the SSC recommendations. Fourthly, it is quite possible that there was latency or a time lead bias between implementation of the education program and any changes in the physicians’ utilization rates. In this study design, we implemented a 1-year gap (2004), between the pre-intervention and post-intervention groups in order to address this issue. In addition, in order to take cohort effects into consideration, we used an identical four year span for the two cohorts, which ought to minimize this issue. However, if we examine the two cohorts as a whole, the post-intervention group is older, has more comorbidities and a higher disease severity than the pre-intervention group; this should result in an underestimation of the benefits of the SSC program to a certain degree.

In conclusion, the implementation of a nationwide education program by a national professional society has a significant impact on physician behavior in sepsis care and this behavior change is associated with a reduction in severe sepsis mortality. The reduction in hospital mortality, despite being statistically significant, is very low and the mortality rate after the intervention is still very high. Clearly there is still much room for improvement and other interventions should be done to improve prognosis of septic patients in Taiwan.

## Supporting Information

Figure S1 **Data processing of the pre-intervention cohort and post-intervention cohort.**
(TIF)

Table S1 **ICD-9-CM codes used to identify bacterial/fungal infections.** The list is adapted from Angus et al in 2001 (*Critical* *Care* *Medicine*, 2001; 29(7): 1303-10).
(DOC)

Table S2 **ICD-9-CM codes suggesting organ failure that is associated with severe sepsis.** The list was first built and validated by Shen et al (*Chest* 2010; 138(2): 298-304).
(DOC)

Table S3 **ICD-9-CM codes not related with severe sepsis.**
(DOC)
