# Supplementary figures and images for: The Impact of Nationwide Education Program on Clinical Practice in Sepsis Care and Mortality of Severe Sepsis: A Population-Based Study in Taiwan
Source: PLoS One. 2013 Oct 4;8(10):e77414. doi: 10.1371/journal.pone.0077414 (PMC3790748; doi:10.1371/journal.pone.0077414)

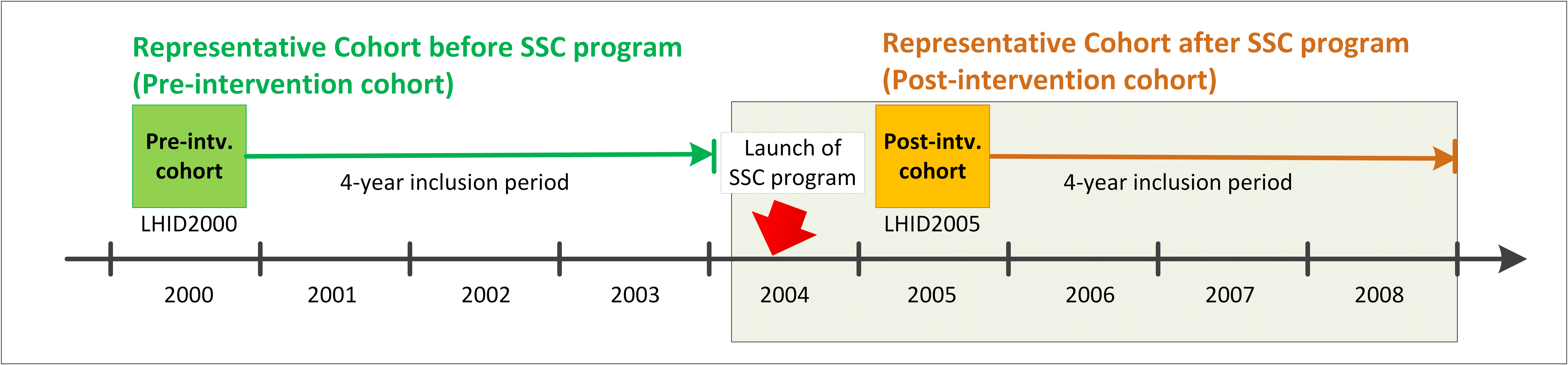

Supplement: Figure S1 — Data processing of the pre-intervention cohort and post-intervention cohort. (TIF) [file pone.0077414.s001.tif]
