# Supplementary material for: The Impact of Nationwide Education Program on Clinical Practice in Sepsis Care and Mortality of Severe Sepsis: A Population-Based Study in Taiwan
Source: PLoS One. 2013 Oct 4;8(10):e77414. doi: 10.1371/journal.pone.0077414 (PMC3790748; doi:10.1371/journal.pone.0077414)
Supplement: Table S1 — ICD-9-CM codes used to identify bacterial/fungal infections. The list is adapted from Angus et al in 2001 (Critical Care Medicine, 2001; 29(7): 1303-10). (DOC) [file pone.0077414.s002.doc]

Table S1. ICD-9-CM codes used to identify bacterial/fungal infections. The list is adapted from Angus et al in 2001. (Critical Care Medicine,2001; 29(7): 1303-10)

| **ICD codes** | **Code description** |
| --- | --- |
| 001.x | Cholera |
| 002.x | Typhoid/paratyphoid fever |
| 003.x | Other salmonella infection |
| 004.x | Shigellosis |
| 005.x | Other food poisoning |
| 008.x | Intestinal infection not otherwise classified |
| 009.x | Ill-defined intestinal infection |
| 010.x | Primary tuberculosis infection |
| 011.x | Pulmonary tuberculosis |
| 012.x | Other respiratory tuberculosis |
| 013.x | Central nervous system tuberculosis |
| 014.x | Intestinal tuberculosis |
| 015.x | Tuberculosis of bone and joint |
| 016.x | Genitourinary tuberculosis |
| 017.x | Tuberculosis not otherwise classified |
| 018.x | Miliary tuberculosis |
| 137.x | Late effect of tuberculosis |
| 020.x | Plague |
| 021.x | Tularemia |
| 022.x | Anthrax |
| 023.x | Brucellosis |
| 024.x | Glanders |
| 025.x | Melioidosis |
| 026.x | Rat-bite fever |
| 027.x | Other bacterial zoonoses |
| 030.x | Leprosy |
| 031.x | Other mycobacterial disease |
| 032.x | Diphtheria |
| 033.x | Whooping cough |
| 034.x | Streptococcal throat/scarlet fever |
| 035.x | Erysipelas |
| 036.x | Meningococcal infection |
| 037.x | Tetanus |
| 038.x | Septicemia |
| 039.x | Actinomycotic infections |
| 040.x | Other bacterial diseases |
| 041.x | Bacterial infection in other diseases not otherwise specified |
| 090.x | Congenital syphilis |
| 091.x | Early symptomatic syphilis |
| 092.x | Early syphilis latent |
| 093.x | Cardiovascular syphilis |
| 094.x | Neurosyphilis |
| 095.x | Other late symptomatic syphilis |
| 096.x | Late syphilis latent |
| 097.x | Other and unspecified syphilis |
| 098.x | Gonococcal infections |
| 100.x | Leptospirosis |
| 101.x | Vincent’s angina |
| 102.x | Yaws |
| 103.x | Pinta |
| 104.x | Other spirochetal infection |
| 110.x | Dermatophytosis |
| 111.x | Dermatomycosis not otherwise classified or specified |
| 112.x | Candidiasis |
| 114.x | Coccidioidomycosis |
| 115.x | Histoplasmosis |
| 116.x | Blastomycotic infection |
| 117.x | Other mycoses |
| 118.x | Opportunistic mycoses |
| 320.x | Bacterial meningitis |
| 321.x | Cryptococcal meningitis |
| 322.x | Meningitis |
| 324.x | Central nervous system abscess |
| 325.x | Phlebitis of intracranial sinus |
| 420.x | Acute pericarditis |
| 421.x | Acute or subacute endocarditis |
| 451.x | Thrombophlebitis |
| 461.x | Acute sinusitis |
| 462.x | Acute pharyngitis |
| 463.x | Acute tonsillitis |
| 464.x | Acute laryngitis/ tracheitis |
| 465.x | Acute upper respiratory infection of multiple sites/not otherwise specified |
| 481.x | Pneumococcal pneumonia |
| 482.x | Other bacterial pneumonia |
| 485.x | Bronchopneumonia with organism not otherwise specified |
| 486.x | Pneumonia |
| 491.21 | Acute exacerbation of obstructive chronic bronchitis |
| 494.x | Bronchiectasis |
| 510.x | Empyema |
| 513.x | Lung/mediastinum abscess |
| 540.x | Acute appendicitis |
| 541.x | Appendicitis not otherwise specified |
| 542.x | Other appendicitis |
| 562.01 | Diverticulitis of small intestine without hemorrhage |
| 562.03 | Diverticulitis of small intestine with hemorrhage |
| 562.11 | Diverticulitis of colon without hemorrhage |
| 562.13 | Diverticulitis of colon with hemorrhage |
| 566.x | Anal and rectal abscess |
| 567.x | Peritonitis |
| 569.5x | Intestinal abscess |
| 569.83 | Perforation of intestine |
| 572.0x | Abscess of liver |
| 572.1x | Portal pyemia |
| 575.0x | Acute cholecystitis |
| 590.x | Kidney infection |
| 597.x | Urethritis/ urethral syndrome |
| 599.0x | Urinary tract infection not otherwise specified |
| 601.x | Prostatic inflammation |
| 614.x | Female pelvic inflammation disease |
| 615.x | Uterine inflammatory disease |
| 616.x | Other female genital inflammation |
| 681.x | Cellulitis |
| 682.x | Other cellulitis or abscess |
| 683.x | Acute lymphadenitis |
| 686.x | Other local skin infection |
| 711.0x | Pyogenic arthritis |
| 730.x | Osteomyelitis |
| 790.7x | Bacteremia |
| 996.6x | Infection or inflammation of device/graft |
| 998.5x | Postoperative infection |
| 999.3x | Infectious complication of medical care not otherwise classified. |
