# Supplementary material for: The Impact of Nationwide Education Program on Clinical Practice in Sepsis Care and Mortality of Severe Sepsis: A Population-Based Study in Taiwan
Source: PLoS One. 2013 Oct 4;8(10):e77414. doi: 10.1371/journal.pone.0077414 (PMC3790748; doi:10.1371/journal.pone.0077414)
Supplement: Table S2 — ICD-9-CM codes suggesting organ failure that is associated with severe sepsis. The list was first built and validated by Shen et al (Chest 2010; 138(2): 298-304). (DOC) [file pone.0077414.s003.doc]

Table S2. ICD-9-CM codes suggesting organ failure that is associated with severe sepsis. The list was first built and validated by Shen et al (*Chest* 2010; 138(2): 298-304).

| **Organ dysfunction** | **Codes** | **Code description** |
| --- | --- | --- |
| Cardiovascular | 458.0  458.8  458.9  785.5  785.51  785.59  796.3 | Hypotension, postural  Hypotension, specified type, NOS  Hypotension, arterial, constitutional  Shock  Shock, cardiogenic  Shock, circulatory or septic  Hypotension, transient |
| Respiratory | 518.81  518.82  518.85  786.09  799.1  96.7x  96.04  93.90 | Acute respiratory failure  Acute respiratory distress syndrome (ARDS)  ARDS after shock or trauma  Respiratory insufficiency  Respiratory arrest  Ventilator management  Endotracheal intubation (emergency procedure)  Continuous positive airway pressure |
| Renal | 580.x  584.x  586  39.95 | Acute glomerulonephritis  Acute renal failure  Renal shutdown, renal failure unspecified  Hemodialysis |
| Hepatic | 570  572.2  573.3  573.4 | Acute hepatic failure or necrosis  Hepatic encephalopathy  Hepatitis (septic & not elsewhere classified)  Hepatic infarction |
| Neurological | 293  348.1  348.3  780.01  780.09  89.14 | Transient organic psychosis  Anoxic brain injury  Encephalopathy, acute  Coma  Altered consciousness, unspecified  Electroencephalography |
| Hematological | 286.2  286.6  286.9  287.3-5  790.92 | Disseminated intravascular coagulation  Purpura fulminans  Coagulopathy  Thrombocytopenia  Abnormal coagulation profile |
| Metabolic | 276.2 | Acidosis, metabolic or lactic |
