# Supplementary material for: The Impact of Nationwide Education Program on Clinical Practice in Sepsis Care and Mortality of Severe Sepsis: A Population-Based Study in Taiwan
Source: PLoS One. 2013 Oct 4;8(10):e77414. doi: 10.1371/journal.pone.0077414 (PMC3790748; doi:10.1371/journal.pone.0077414)
Supplement: Table S3 — ICD-9-CM codes not related with severe sepsis. (DOC) [file pone.0077414.s004.doc]

Table S3. ICD-9-CM codes not related with severe sepsis.

|  | Pre-intervention cohort (2000-2003) | |  | Post-intervention cohort (2005-2008) | |
| --- | --- | --- | --- | --- | --- |
| Diagnosis | n=14848 | (%) |  | n=24858 | (%) |
| Cardiovascular syphilis | 0 | (0.00) |  | 1 | (0.00) |
| Central nervous system tuberculosis | 26 | (0.18) |  | 20 | (0.08) |
| Congenital syphilis | 1 | (0.01) |  | 0 | (0.00) |
| Dermatomycosis not otherwise classified or specified | 7 | (0.05) |  | 8 | (0.03) |
| Dermatophytosis | 63 | (0.42) |  | 70 | (0.28) |
| Early symptomatic syphilis | 1 | (0.01) |  | 1 | (0.00) |
| Genitourinary tuberculosis | 6 | (0.04) |  | 7 | (0.03) |
| Gonococcal infections | 0 | (0.00) |  | 1 | (0.00) |
| Intestinal tuberculosis | 16 | (0.11) |  | 17 | (0.07) |
| Late effect TB | 265 | (1.78) |  | 411 | (1.65) |
| Leprosy | 0 | (0.00) |  | 6 | (0.02) |
| Late syphilis latent | 1 | (0.01) |  | 0 | (0.00) |
| Melioidosis | 1 | (0.01) |  | 3 | (0.01) |
| Miliary tuberculosis | 13 | (0.09) |  | 27 | (0.11) |
| Neurosyphilis | 0 | (0.00) |  | 3 | (0.01) |
| Other and unspecified syphilis | 15 | (0.10) |  | 18 | (0.07) |
| Other bacterial zoonoses | 1 | (0.01) |  | 4 | (0.02) |
| Other mycobacterial disease | 8 | (0.05) |  | 46 | (0.19) |
| Other spirochetal infection | 1 | (0.01) |  | 0 | (0.00) |
| Tetanus | 2 | (0.01) |  | 2 | (0.01) |
| Tuberculosis of bone and joint | 20 | (0.13) |  | 23 | (0.09) |
| Total | 447 | (3.0) |  | 668 | (2.7) |
